# Supplementary material for: Biomechanical evaluation of unilateral subcondylar fracture of the mandible on the varying materials: A finite element analysis
Source: PLoS One. 2020 Oct 8;15(10):e0240352. doi: 10.1371/journal.pone.0240352 (PMC7544122; doi:10.1371/journal.pone.0240352)
Supplement: Attachment — Submitted filename: Reviewer 2_Point-By-Point Response.docx [file pone.0240352.s001.docx]

**POINT-BY-POINT RESPONSE FORM**

**Biomechanical evaluation of unilateral subcondylar fracture of the mandible on the varying materials: a finite element analysis**

**#Reviewer2**

**Comments and suggestions**

Summary of the research and my overall impression:

The aim of this research is particularly relevant to the field of Craniomaxillofacial Surgery because it is related to oral fractures, more specifically to mandible condylar ones. This subject is really challenging. Sometimes, surgeons don’t choose the open access to treat these fractures either due to anatomic reasons because important structures can be found there or due to the difficulty to reduce and to fix the fractures.

It is known that the open treatment is better than the closed one because it can facilitate the return to normal activities such as mastication and occlusion as soon as possible. Besides that, some complications caused by the non-surgical treatment can be avoided.

This paper shows a study about four kinds of ORIF to treat the mandible subcondylar fractures: titanium (Ti), magnesium alloy (Mg alloy), biodegradable polymers such as poly-L-lactic acid (PLLA) and a hydroxyapatite/poly-L-lactide (HA-PLLA). It is justified by the increasing demand of re-absorbable materials to be used instead of titanium, which is the gold standard material, owing to some disadvantages.

The methodology is especially interesting, based on other studies that have been published in high impact factor journals.

**Abstract:**

In order to be better understood by the readers of the journal, it would be interesting to follow the acronyms with the complete name of the materials. For example ti: titanium.

I suggest removing this sentence: “Deformation of HA-PLLA was approximately 0.15 mm upon 300 N of masticatory loading”. It is hard to understand in this section and it is well explained in the results section.

Response: Thank you for your suggestion. We have written Ti as titanium and removed the sentence “Deformation ~ loading.”

- Line 18-21 : Here, we simulated the mechanical properties of four fixation materials, namely, Titanium (Ti), Magnesium-alloy (Mg-alloy), poly-L-lactic acid (PLLA), and hydroxyapatite/poly-L-lactide (HA-PLLA), in a finite element analysis model of subcondylar fracture.

- Line 29 : We have removed this sentence

**Introduction:**

Line 10: “Subcondylar fractures can be treated conservatively or surgically”.

What does “conservatively” mean? The best term may be “non-surgically”, as we can have a conservative surgery, for example, depending on our care during the procedure.

Response: We revised the introduction section.

- Line 32-44 : The subcondylar fracture commonly occurred at the sigmoid notch of mandible. Although, surgical approaches for open reduction risk facial nerve injury, it presented superior results to and fewer complications than closed reduction [1], for which present feasible visual field for accurate reduction with internal fixation devices such as mini-plates and screws [2]. However, the subcondyle, in particular, is a very difficult to be surgically approached that only present limited surgical access due to the presence of facial nerve. Therefore, surgeons’ experience and the fixation materials should be carefully considered for a successful clinical outcome. For the fixation materials, that requires mechanical strength as well as biocompatibility. Recently, with development of biomaterials for surgical treatment of mandible fractures, composites of unsintered hydroxyapatite particles and poly-l-lactide (u-HA/PLLA) has been used for open reduction, which reported reliable clinical outcome in mandibular body fracture [3].

**Materials and methods:**

In Figure 1, the posterior plate does not seem to be completely attached to the bone. I would like you to confirm this and try to explain it better.

The second screws (considering the superior to inferior) are closer to the fracture line than the ideal. On the anterior plate, this screw seems to be in the fracture line. Depending on the direction of the perforation to install this screw, I think that the fracture can be “open”. Maybe, this fact can be considered a bias.

I don’t think it can influence the results, but I think that you can try to reinstall this plate, virtually.

I suggest you provide more information about the masticatory load that was applied. Why did you choose this load? Isn´t it too high? I would like to see other papers that justify this choice.

Response: Thank you for your comments. As you suggested, we have repositioned the plate position and re-performed the analysis. Please find Figure 2 and Figure 5 for the plate position.

Also, We set up occlusal forces 132N at one week and 300N at 6 weeks after surgery. One week and 6 weeks after surgery are important timing in mandible surgery, for patients would commonly start to have food intake under elastic occlusal guidance. In healthy adult, usual mastication force is 700N (that was explained Line 234-243). Clinically relevant mastication force during formation of primary bony callus in patients who had mandible surgery usually would be observed from one week to 6 weeks after surgery, though we simulated deformation of materials to extreme condition such as 1000N.

These set up based on other previous studies as below. (we’ll added these two references in revised manuscript).

J Oral Maxillofac Surg

. 2000 Apr;58(4):370-3; discussion 373-4. doi: 10.1016/s0278-2391(00)90913-3.

Measure of bite force and occlusal contact area before and after bilateral sagittal split ramus osteotomy of the mandible using a new pressure-sensitive device: a preliminary report

K Harada 1, M Watanabe, K Ohkura, S Enomoto

J Oral Maxillofac Surg

. 2014 Feb;72(2):402.e1-13. doi: 10.1016/j.joms.2013.10.003. Epub 2013 Oct 17.

Biomechanical evaluation of magnesium-based resorbable metallic screw system in a bilateral sagittal split ramus osteotomy model using three-dimensional finite element analysis

Jin-Yong Lee 1, Jung-Woo Lee 2, Kang-Mi Pang 3, Hyoun-Ee Kim 4, Soung-Min Kim 5, Jong-Ho Lee 6

- Line 234-236 : We assumed 132 N of force at 1 week after surgery, 300 N at 6 weeks after surgery, and 700 N in the healthy adult’s mastication loading based on previous studies [4,27,28].

**Results:**

In Figure 2: “middle column, stress on fixations (mini-plates and screws) but we can just see the plates, without screws”.

I think that you need to change this caption due to the fact that the screws are not there, just the plates.

Do you have information about the bone? If so, you can insert main maximum tension and minimum tension to increase the amount of information and to make this article even more relevant.

Response: As suggested, we have added maximal and minimal tensile stress values and revised the middle column in Fig 3.

- Line 144-152 : The tensile stress distribution of cortical bone was highest for Mg-alloy, followed by Ti, HA-PLLA and PLLA. The stress distribution was similar between Ti and Mg-alloy and between HA-PLLA and PLLA (Table 2 and Table 3). On the other hand, the tensile stress distribution of cancellous bone was highest for Mg-alloy, followed by HA-PLLA, Ti and PLLA. The stress distribution of screw hole in the bones was higher in the anterior screw hole compared to the posterior hole. Facing the fracture line, the contact area of the screw hole was applied compressive stress, and tensile stress occurred on the opposite side (Fig 3a,d,g,j).
