# Supplementary material for: Biomechanical evaluation of unilateral subcondylar fracture of the mandible on the varying materials: A finite element analysis
Source: PLoS One. 2020 Oct 8;15(10):e0240352. doi: 10.1371/journal.pone.0240352 (PMC7544122; doi:10.1371/journal.pone.0240352)
Supplement: Attachment — Submitted filename: Response to Reviewers.docx [file pone.0240352.s002.docx]

**POINT-BY-POINT RESPONSE FORM**

**Biomechanical evaluation of unilateral subcondylar fracture of the mandible on the varying materials: a finite element analysis**

**#Reviewer1**

**Comments and suggestions**

Reviewer #1: I here carefully re-evaluated the revised set of the manuscript and found it no merits of publication. I'm sorry but I can't support this study article. Further the re-submitted article would be wrongly carried with English grammatical corrections.

Response: Thank you for your suggestion.

We have undergone the service by Editage (www.editage.co.kr) for English language editing. I attached the certification.

Moreover, our study examined simulations of four different fixation materials (titanium, magnesium alloy, poly-L-lactic acid [PLLA], and hydroxyapatite/poly-L-lactide [HA-PLLA]) in finite-element analysis models of subcondylar mandibular fracture.

Predicted result using computer simulation (mathmetical annalysis) was affected only by change of material’s property (Elastci Modulus, Poisson’s Ratio) under same conditions (design, fracture type, load condition etc.). therefore, repeated simulation result is always same for same material with same condition.

Reviewer #2: Thanks for answering my questions. I think that the information now is better and you improved your paper. I appreciate it.

Response: Thank you for your kind comment.
